# Recall of Reverberant Speech in Quiet and Four-Talker Babble Noise

**DOI:** 10.3390/brainsci11070891

**Published:** 2021-07-05

**Authors:** Miseung Koo, Jihui Jeon, Hwayoung Moon, Myung-Whan Suh, Jun-Ho Lee, Seung-Ha Oh, Moo-Kyun Park

**Affiliations:** 1 Department of Otorhinolaryngology-Head and Neck Surgery, Seoul National University Hospital, Seoul 03080, Korea; misng9@gmail.com (M.K.); drmung@daum.net (M.-W.S.); junlee@snu.ac.kr (J.-H.L.); shaoh@snu.ac.kr (S.-H.O.); 2 Yeongeon Medical Campus, Seoul National University College of Medicine, Seoul 03080, Korea; wlgml0720@snu.ac.kr (J.J.); hymun1372@snu.ac.kr (H.M.); 3 Sensory Organ Research Institute, Seoul National University Medical Research Center, Seoul 03087, Korea

**Keywords:** reverberation, noise, hearing, speech intelligibility, listening effort

## Abstract

Using behavioral evaluation of free recall performance, we investigated whether reverberation and/or noise affected memory performance in normal-hearing adults. Thirty-four participants performed a free-recall task in which they were instructed to repeat the initial word after each sentence and to remember the target words after each list of seven sentences, in a 2 (reverberation) × 2 (noise) factorial design. Pupil dilation responses (baseline and peak pupil dilation) were also recorded sentence-by-sentence while the participants were trying to remember the target words. In noise, speech was presented at an easily audible level using an individualized signal-to-noise ratio (95% speech intelligibility). As expected, recall performance was significantly lower in the noisy environment than in the quiet condition. Regardless of noise interference or reverberation, sentence- baseline values gradually increased with an increase in the number of words to be remembered for a subsequent free-recall task. Long reverberation time had no significant effect on memory retrieval of verbal stimuli or pupillary responses during encoding.

## 1. Introduction

Hearing-impaired (HI) people often report difficulty in comprehending speech in noisy or reverberant environments, whereas young adults with normal-hearing (NH) can reportedly cope with reverberant speech with a moderate amount of background noise [1,2]. HI elderly and children are more vulnerable to the effects of reverberation than NH elderly and children [3]. The Framework for Understanding Effortful Listening (FUEL) [4] defines listening effort as “the deliberate allocation of mental resources to overcome obstacles in goal pursuit when carrying out a [listening] task”, and suggests that signal-to-noise ratio (SNR) and reverberation time (RT) as transmission factors and indirect inputs via the cognitive capacity component. Preceding sounds, if reverberant and slowly decaying, are likely to mask successive sounds, making it harder to understand speech. Long RT is detrimental to perceived speech quality; thus, speech intelligibility is highly influenced by SNR and RT [5]. Moreover, reverberation distorts the signals reaching the ear and impedes selective auditory attention [6], and this detrimental impact on speech intelligibility and listening effort can be measured by speech transmission index [7,8,9], electro-dermal activity levels, and subjective ratings [10]. Xia et al. (2018) [11] reported that both NH and HI listeners found it hard to recognize words in noise, showing degraded performance as a result of increased reverberation time.

Behavioral analysis, e.g., a dual-task paradigm, has been widely used to study the impact of noise interference in listening speech or reverberation on memory. Among such studies, Ng et al. (2013, 2015) [12,13] have developed a dual-task paradigm (i.e., sentence-final word identification and recall, SWIR), involving a free recall (later validated in Sweden and Denmark [14]), where HI participants have to listen and memorize a list of sentences heard along with background noise. The identification task requires repetition of a sentence after the presentation, whereas the recall task requires free recall of as many sentences as possible after a series of sentences. Ng et al. (2015) [12] revealed that HI listeners benefited from a noise reduction scheme, which improved recall regardless of working memory (WM) capacity. In particular, listeners with low WM capacity tended to recall more words late in the list than in other positions [12]. Yet, only a few studies have investigated the effects of reverberation on memory or cognitive load. Kjellberg (2004) [15] addressed theoretical aspects of the unfavorable impact of reverberation on the cognitive processing of speech and predicted that high cognitive demand or fatigue would reduce the WM performance of the processing of speech degraded by long RT because perception of the inputs relies on a top-down and resource-demanding process. A later experimental study supported the prediction that NH subjects recall fewer words at longer RT when there was background noise [16].

Pupillometry has been reported as a physiological measure that reliably indicates cognitive load or effort in performing tasks [17,18]. An early study by Beatty and Kahneman (1966) [19] addressed the possibility that pupillary response is sensitive to the momentary load and reported that the pupil dilates more steeply in the long-term memory condition (recall of familiar telephone numbers) than in the short-term memory condition (recall of unfamiliar telephone numbers). Our previous study [20] used a relative baseline correction for the pupil data collected from test subjects who listened to a set of seven sentences presented one after another. In this correction method we took a 1 s pre-stimulus baseline pupil size as a baseline for the initial stimulus in a set and computed the baseline and dilation for each sentence throughout the list. We found that the sentence baseline increased consecutively in line with the increase in memory load (number of words to be recalled). Using the same correction method, a similar study by Bönitz et al. (2021) [21] analyzed intercept and slope differences in sentence-baselines and dilations to compare three recall conditions with different list lengths (three or six words in a list) and two no-recall conditions. They observed that sentence baseline steeply increased under recall but declined under no-recall conditions. Moreover, they linked reduced sentence dilation to lower listening effort under noise reduction.

The present study was motivated by the idea that more effort allocated to listening due to adverse listening conditions may reduce the cognitive resources allocated to memory. A cognitive-behavioral measure (i.e., free recall of spoken words), along with a physiological measure (i.e., changes in pupil dilation), were used to estimate listening effort or cognitive capacity allocation during encoding and to explore whether a similar response was elicited in the two measures. In addition, we investigated the following hypotheses: (1) speech with long RT may negatively affect recall performance in NH adults, even though speech is presented at a near-ceiling level, (2) speech in a noisy listening environment may negatively affect recall performance in NH adults, even though speech is presented at a near ceiling level, (3) recall performance may depend on the target word’s position in the list (i.e., serial-position effect), which will be explained in the following section, (4) pupil dilation response to each stimulus may tend to increase or decrease as a function of stimulus presentation order due to the increases in memory load and/or listening effort, and (5) these trends may be affected by the RT and/or background noise.

## 2. Materials and Methods

### 2.1. Subjects

Thirty-four NH adults, 18 females, mean age = 28.5 years, SD = 5.7, age range: 20–39, who had no history of middle ear disease, eye problems, cognitive problems, neurological diseases, dyslexia, or diabetes mellitus were recruited. They were recruited by advertisement in the university community at Seoul National University College of Medicine. Hearing loss was defined as a pure-tone average of 0.5, 1, 2, and 4 kHz of 20 dB HL in the poor ear. The binaural average of pure-tone thresholds at the four frequencies was 4.6 dB HL (SD = 4.1). The study was approved by the Seoul National University Hospital Institutional Review Board (1805-065-946). All subjects provided written informed consent prior to participation.

Cognitive function was assessed using the Korean version of the Montreal Cognitive Assessment (MoCA-K) [22] by evaluating performance in various cognitive domains: short-term memory, visuospatial or executive functioning, phonemic fluency, verbal abstraction, attention, concentration, WM, language, and orientation to time and place. Scores in the MoCA-K range from 0 to 30, with a cutoff score of 23. The enrolled participants had an average score of 28.6 (SD = 1.7). All the participants were assessed in terms of hearing and cognitive function by means of otoscopy, pure-tone audiometry, and MoCA-K.

### 2.2. Test Materials

The test materials for the free recall task consisted of 14 sets of 7 sentences each, including 4 lists used for practice. These sentences were selected from the Korean Hearing in Noise Test (HINT) sentences [23], which consists of 12 lists of 20 sentences each, following the stimulus selection method described in SWIR studies [12,14]. The HINT test prior to the recall task was used for calculating SNRs in the noise conditions. The average HINT SNR values for the 3 noise conditions were 3.63 (SD = 1.09) in anechoic condition, 8.3 (SD = 1.52) in the short-RT condition, and 15.41 (SD = 3.72) in the long-RT condition. The test items in each list were categorized into 3 types, as described in Ng et al. (2015) [12] and Lunner et al. (2016) [14]: The first and second words were assigned to the ‘primary’ position, the third to fifth words to the ‘asymptote’ position, and the sixth and seventh words to the ‘recency’ position.

Four-talker babble (4T), recorded from 4 native Korean speakers (2 males and 2 females), was presented as background noise. The noise was post-filtered to resemble the long-term average spectrum of the target sentences used for the recall task. The 4T noise started 3 s before the onset of a sentence and ended 3 s after sentence offset.

### 2.3. Design

This is a follow-up study of our previous work [20]. We used 4 different test conditions (2 noise conditions × 2 RT conditions), simulated in MATLAB, to assess the effects of noise and RT (short and long) on memory and pupillary response (Table 1). The test conditions and the sentence lists used in each condition were randomized to remove order effects. Participants performed only the recall task, and did not perform the identification task during the pupil-size recording. After 7 sentences, participants were encouraged to recall the initial words of as many sentences as possible, in any order. We used the results of the recall test to determine the average score in each listening condition and the list position of the recalled words. To evaluate serial-position effects, sentences in each list were allocated as follows: the first and second sentences to the primacy, third to fifth sentences to the asymptote, and sixth to seventh sentences to the recency list position.

### 2.4. Procedure

To avoid the effect of subsequent fatigue on pupillary response [24], participants had a 2–3 min rest between trials of HINT and of the test, including identification and recall tasks. Pupil size was recorded only when the participants were listening to the lists of sentences. These tests were performed in a soundproofed acoustic booth. Total testing time was approximately 90 min, including 10 min of break time.

**Figure 1:**
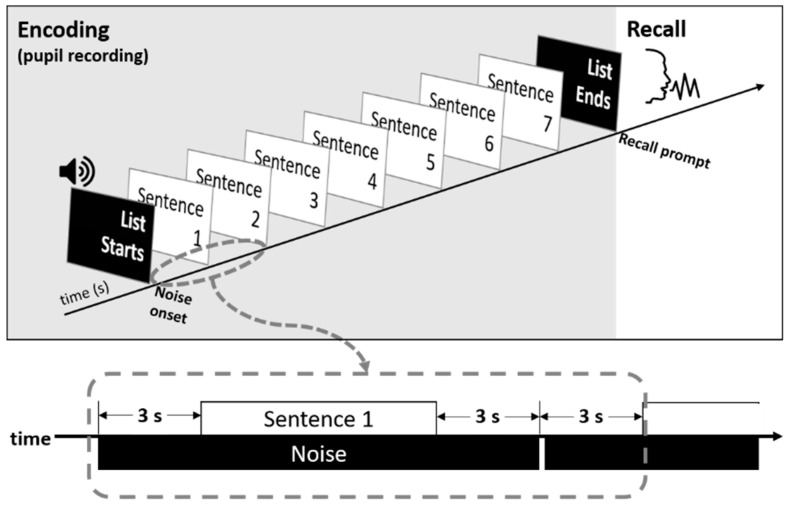
Overview of experimental procedures used in this study.

Figure 1 shows an overview of the experimental design used in this study.

The 2 simulated reverberation conditions were designed to reproduce the listening environments experienced by most patients in the Seoul National University Hospital [25]. The RT for each condition is shown in Table 2. In reverberant conditions, the chosen level of reverberation was also applied to the 4T. After stimulus presentation (i.e., a set of 7 sentences), a short beep sound was presented to prompt listeners to recall the words they heard and remembered.

In total, 40 HINT sentences were presented to each participant; 20 sentences were repeated for each noise condition (conditions 3 and 4 in Table 1). Participants sat in a chair with a single loudspeaker (Genelec 8040B, Iisalmi, Finland) located at ear height, 1 m in front of them. For each condition, they were required to repeat each sentence immediately after listening to it and using the HINT adaptive method for speech reception threshold measurement. The level of target speech was adjusted depending on the participant’s response, while the HINT noise was maintained at 60 dB SPL. During the HINT, for the first 4 sentences out of 20 sentences in each list, the SNR was decreased by 2 dB if the participant repeated the sentence correctly, and increased by 6 dB if the participant repeated the sentence incorrectly. From sentence 5 onward, the SNR was decreased by 1 dB after correct responses and increased by 3 dB after incorrect responses. The results were used to calculate an individualized SNR that predicted 80% speech perception.

### 2.5. Identification and Recall Tasks

The 2 tasks used in this study were modified from [12,13,14]: the identification task (repeating the final word immediately after listening to each sentence), and the free-recall task (recalling the words in any order). However, most Korean sentences tended to end with a predicate. Therefore, participants in this study were required to repeat and recall the initial word of each sentence.

Each participant completed 4 training lists and 10 test lists of 7 sentences each. Training sessions were performed in the 2 noise conditions using the initially calculated SNR corresponding to 80% speech perception during the training session that instructed the participants to repeat the initial word of each sentence. After each list of 7 sentences, they were asked to recall as many target words as they could, in any order. The masker level was maintained when 6 or 7 words were recalled correctly, decreased by 1 dB when 4 or 5 words were recalled correctly, and decreased by 2 dB when 0–3 words were recalled correctly. This adjustment, depending on the participant’s identification score, was needed to reach individual SNR corresponding to 95% speech perception. For testing, the lists of test sentences was presented at individualized SNRs that predicted 95% speech perception for each participant. In each condition, the volume of the sentence was fixed at 60 dB and the volume of 4T was adjusted.

### 2.6. Pupillometry

Pupil diameter data were recorded using an eye-tracker (Pupil Labs, Berlin, Germany) with 200 Hz binocular cameras (1920 × 1080 pixels, 120 Hz sampling frequency on a subset of 320 × 280 pixels) connected to a PC via MATLAB software to store the data in real time. Pupil diameter was typically measured in the left eye. When measurements in the left eye were of poor quality, it was measured in the right eye. Pupil dilation data from an eye (left or right) were included in the analysis because we were unsure how to prove collinearity in data between left and right eyes.

The instrument used infrared video-based tracking technology to measure pupil diameter. The spatial resolution of the pupillometer was 200 × 200 pixels. The beginning of each stimulus was measured manually on the audio recording. Participants were instructed to wear a glasses-type eye-tracker and focus on a fixation dot positioned 2 m away. Breaks were given between tests. During the procedure, the experimenter checked the validity of the pupil data and took corrective action, e.g., giving proper instruction to reduce excessive blinking, if necessary.

The room’s illumination was adjusted so that pupil diameter was in the middle of each individual’s dynamic range. Pupil diameter was measured at maximum illumination (250 lx) and in darkness. These adjustments in illumination prevented ceiling and floor effects in the pupil dilation response. The mean room illumination after individual adjustment was 92 ± 34 lux. Participants were encouraged to avoid the use of eye make-up and particularly eyeliner, which can be mistaken for the pupil by the pupillometer [26]. Contact lenses were permitted, as they did not impede capture of the pupil diameter.

The median absolute deviation method of Kret and Sjak-Shie, [27] was used for blink detection. In addition, data points whose values were more than 2.5 standard deviations from the mean diameter were coded as blinks. The data that contained more than 25% of blinks from the onset of noise until 6.5 s after sentence completion were discarded. The blink interval was replaced by linear interpolation beginning 50 ms before and ending 150 ms after a blink. The data were passed through a 5-point moving average smoothing filter. The length of the window of the filter, which corresponds to the time range under one side of the rectangular window, was 0.4 s. Divisive baseline correction was used to generate pupil data for each condition.

We used two measurements of pupil dilation: (1) baseline pupil size and (2) peak pupil dilation (PPD). The PPD was measured at 3.5 s from sentence onset. The average pupil diameter in the 1 s preceding the start of speech was used as a baseline. The PPD was calculated using divisive baseline correction relative to the average curve of the seven sentences.

### 2.7. Statistical Analysis

Statistical software (Prism 9.1.0, GraphPad Software, Inc. La Jolla, CA, USA) was used for the analysis of recall performance. Recall percentage results were analyzed using a 3-way ANOVA repeated-measures model (independent variables: background noise, RT, serial position), followed by a Tukey multiple comparison test.

SPSS 25 statistical software (SPSS Inc., Chicago, IL, USA) was used for the analysis of pupil response data. This data was analyzed using linear mixed models (LMMs), following the same statistical method as in Ohlenforst et al. (2018) [28], to compare the fixed effects of RT, noise, and stimulus presentation order on the pupil diameter data. The fixed effects included the categorical variables. Post hoc pairwise comparisons were Bonferroni corrected. After preprocessing, the pupil data from 25 out of 34 participants were considered valid because of large numbers of missing or erroneous data. Of the 450 pupil traces, 20 (4.44%) were identified as invalid.

## 3. Results

### 3.1. Recall Scores

**Figure 2:**
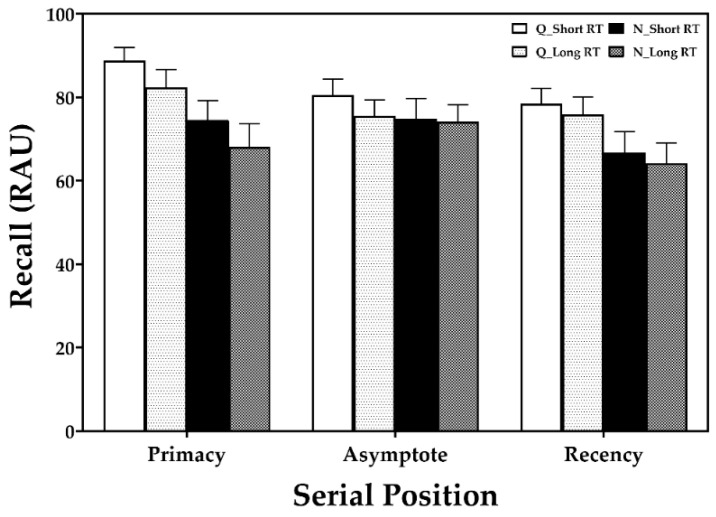
Recall scores in the quiet condition versus four-talker babble noise with short or long reverberation time (RT). Error bars represent ±1 standard error of the mean. Note: Q, quiet condition; N, noise condition; RAU, rationalized arcsine unit.

The percentage of correctly recalled words in each listening condition is shown in Figure 2. The 3-way ANOVA with repeated measures revealed a significant main effect of noise [F (0.8094, 26.71) = 22.29, *p* = 0.0002] on recall performance, indicating that listeners recalled fewer words due to the noise interference. The listeners also demonstrated decreased performance in the long-RT condition compared to the short-RT condition, although the difference was not significant. Although no significant effect of serial position on recall performance was found, a serial position curve seemed to be present in the absence of background noise with both RTs. 

### 3.2. Pupil Data: Baseline and Peak Pupil Dilations

**Figure 3:**
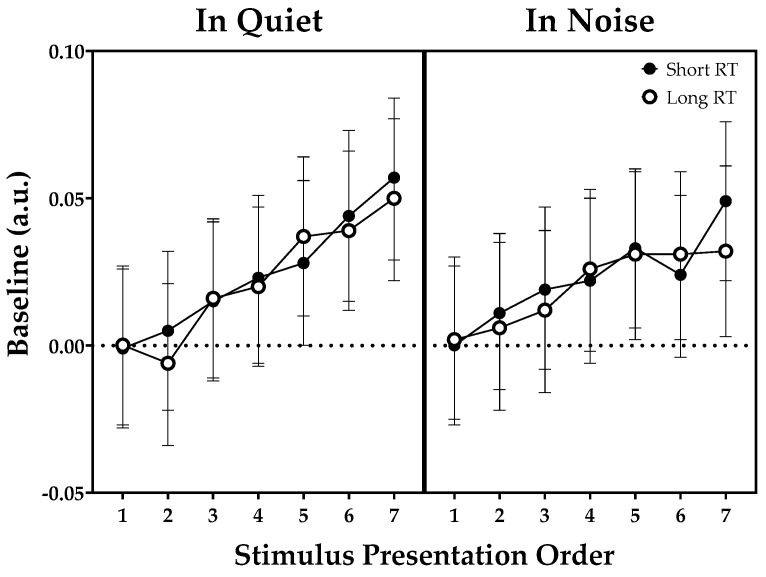
Sentence-baseline values in the quiet condition versus four-talker babble noise as a function of stimulus presentation order. Error bars are 95% confidence intervals for the mean. Note: RT, reverberation time.

Baseline in the quiet condition versus 4T noise as a function of stimulus presentation order (short or long RT) are shown in Figure 3. The LMM on the baseline values demonstrated a significant fixed effect of stimulus presentation order, indicating an increase in sentence baseline relative to the initial sentence baseline with the increase in memory load (number of items to be remembered). No other significant effects were found.

**Figure 4:**
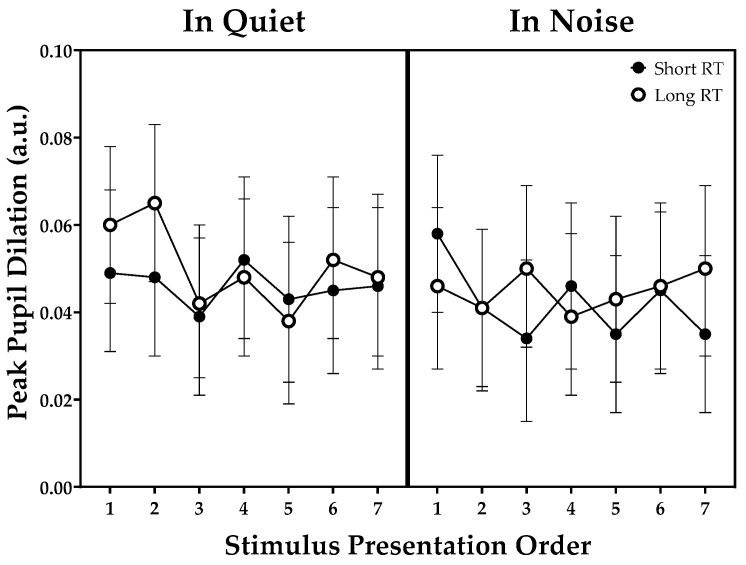
Peak pupil dilation values in the quiet condition versus four-talker babble noise as a function of stimulus presentation order. Error bars are 95% confidence intervals for the mean. Note: RT, reverberation time.

PPD values in the quiet condition versus four-talker babble noise as a function of stimulus presentation order are shown in Figure 4. The LMM on the peak pupil dilation values found no significant effects among the three factors (stimulus presentation order, noise, and RT) mentioned earlier.

## 4. Discussion

In this study, which extends our prior work [20], we focused on the unfavorable effects of background noise and/or prolonged RT on recall performance, as well as pupil dilation responses for each stimulus in a list during the encoding period in a group of NH listeners. We found impaired-free recall of spoken words due to the noise present in the signal. In addition, the pupil dilation baselines consecutively increased as the number of words to be recalled increased during the encoding phase.

Regarding hypothesis 1, long RT had no significant effect on recall performance, although it tended to reduce performance in comparison with short RT (Figure 2), unlike in the study by Ljung and Kjellberg [16]. The difference may be attributed to different acoustic configurations used in the two studies. Ljung and Kjellberg [16] used broadband noise and reverberation produced by multiple loudspeakers. In addition, they used a specified SNR of 15 dB for all the participants, whereas the current study used individualized SNR equivalent to 95% speech perception determined for each participant. They also assigned an additional task that had the participants guess which category the sentence belonged in out of a total of 20 sentences grouped into two categories (short and long RT conditions). Kuusinen et al. (2020) [29] also found no significant difference in speech recognition thresholds influenced by reverberation. The discrepancy might be originated from source-listener distance (typically 1 m).

In the present study, NH listeners found it more difficult to recall speech presented in competing speech noise than in the quiet condition. We tested the combined effect of noise and room reverberation on recall performance and observed significantly degraded performance in the noise condition compared with the quiet condition (Figure 2). Thus, hypothesis 2 was confirmed, and the data indicate that background noise interferes with how listeners encode and retrieve memory items presented auditorily, contrary to our previous finding that background noise did not significantly affect recall performance [20]. However, we note that the previous experiment did not include any reverberation. Similar to the present study, Sarampalis et al. (2009) [30], which assessed NH listeners’ word-memory performance using eight-sentence lists, found a negative impact of background noise on both speech intelligibility and free-recall performance. Other similar studies, conducted on HI listeners, showed a reduction in recall performance by noise without a noise reduction scheme [12,13,14]. However, these studies collected data from HI listeners with symmetric bilateral hearing loss and their primary task was to memorize sentence-final words instead of sentence-first words.

As to hypothesis 4, there was an increasing trend in sentence-baseline values with an increase in the number of items to be remembered in a list (Figure 3). This finding is in line with our previous study performed on a group of NH listeners in quiet versus 4T conditions without any reverberation [20]. Consistent with the findings of Beatty and Kahneman (1966) [19] and Bönitz et al. (2021) [21], the increasing trend in sentence baseline can be interpreted as increasing memory effort. In addition to the relative baseline correction used in this study, the latter study [21] analyzed the intercept and slope of sentence baseline and sentence dilation. The authors concluded that changes in baseline may reflect the participants’ expectation about test difficulty even before the task was given. However, with regard to hypothesis 5, we found no statistically significant changes in pupil dilation responses, both sentence baselines and PPD, in the presence of either background noise or reverberation. In contrast to our initial assumption that more effort needed to listen due to interfering noise or reverberation would lead to significant changes in the pupil dilation responses, our findings revealed no such outcome. Whereas many studies on speech recognition have revealed that effortful listening can affect pupil responses simultaneously recorded during the repetition period, further studies based on a more effective analysis method are required to uncover the link between increased effort in listening and decreased memory.

Further study is required to investigate the influence of reverberation on memory performance with a better test environment (e.g., reverberant field or multiple loudspeakers), instead of processed stimuli. Greater effort to find pupillometry parameters for analysis that can associate with behavioral cognitive results is also needed.

## Figures and Tables

**Table 1:** Reverberation time and noise (4T babble) conditions used in testing.

Test Condition	Noise	Reverberation
**1**	Quiet	Short RT
**2**	Quiet	Long RT
**3**	4T noise	Short RT
**4**	4T noise	Long RT

RT: Reverberation time.

**Table 2:** Reverberation time (seconds) for reverberant conditions.

Condition	Frequency (Hz)					
	125	250	500	1k	2k	4k
**Short RT**	0.63	0.49	0.53	0.57	0.55	0.50
**Long RT**	2.3	1.89	1.83	1.70	1.52	1.27

## Data Availability

The data presented in this study are available on request from the corresponding author. The data are not publicly available due to participant privacy.
